# Effects of prone and lateral positioning alternate in high-flow nasal cannula patients with severe COVID-19

**DOI:** 10.1186/s13054-022-03897-2

**Published:** 2022-01-25

**Authors:** Yang Chong, Chuanchuan Nan, Wenjing Mu, Changsong Wang, Mingyan Zhao, Kaijiang Yu

**Affiliations:** 1 Department of Critical Care Medicine, The First Affiliated Hospital of Harbin Medical University, No.23 Youzheng Street, Harbin, 150001 Heilongjiang Province China; 2 Department of Critical Care Medicine, Harbin Medical University Cancer Hospital, No.150 Haping Road, Harbin, 150001 Heilongjiang Province China; 3 Department of Critical Care Medicine, Shenzhen People’s Hospital, The Second Clinical Medical College of Jinan University, The First Affiliated Hospital of Southern University of Science and Technology, No.1017 Dongmen North Road, Shenzhen, 518000 Guangdong Province China

To date, the COVID-19 pandemic remains widespread globally, placing a heavy burden on healthcare systems around the world. High-flow nasal cannula (HFNC) improves oxygenation and reduces the need for endotracheal intubation in comparison with standard oxygen therapy in patients with severe COVID-19 [1]. During HFNC treatment, the prone position is associated with a significant benefit on oxygenation, but the low compliance of awake patients limits the clinical application of the prone position [2]. The lateral position may also be associated with beneficial effects of gas exchange, especially in unilateral lesions [3]. Although there are studies evaluating the efficacy of both prone and lateral positioning [4, 5], comparative studies on the efficacy of prone and lateral positions in HFNC patients with severe COVID-19 are rarely reported.

In this single-center prospective study, a total of 10 severe COVID-19 patients were included in the treatment center for severe COVID-19 patients in Heilongjiang Province of China. The severe COVID-19 patients in our study were those admitted to the ICU with PaO2/FiO2 ≤ 300 mmHg. Patients in supine position (0.5 h), left lateral position (1.5 h), supine position (0.5 h), right lateral position (1.5 h), supine position (0.5 h) , and prone position (1.5 h) treatment sequence. Blood oxygen saturation and respiratory rate were observed at each time point, and blood gas analysis was performed. (Fig. 1).

**Fig. 1:**
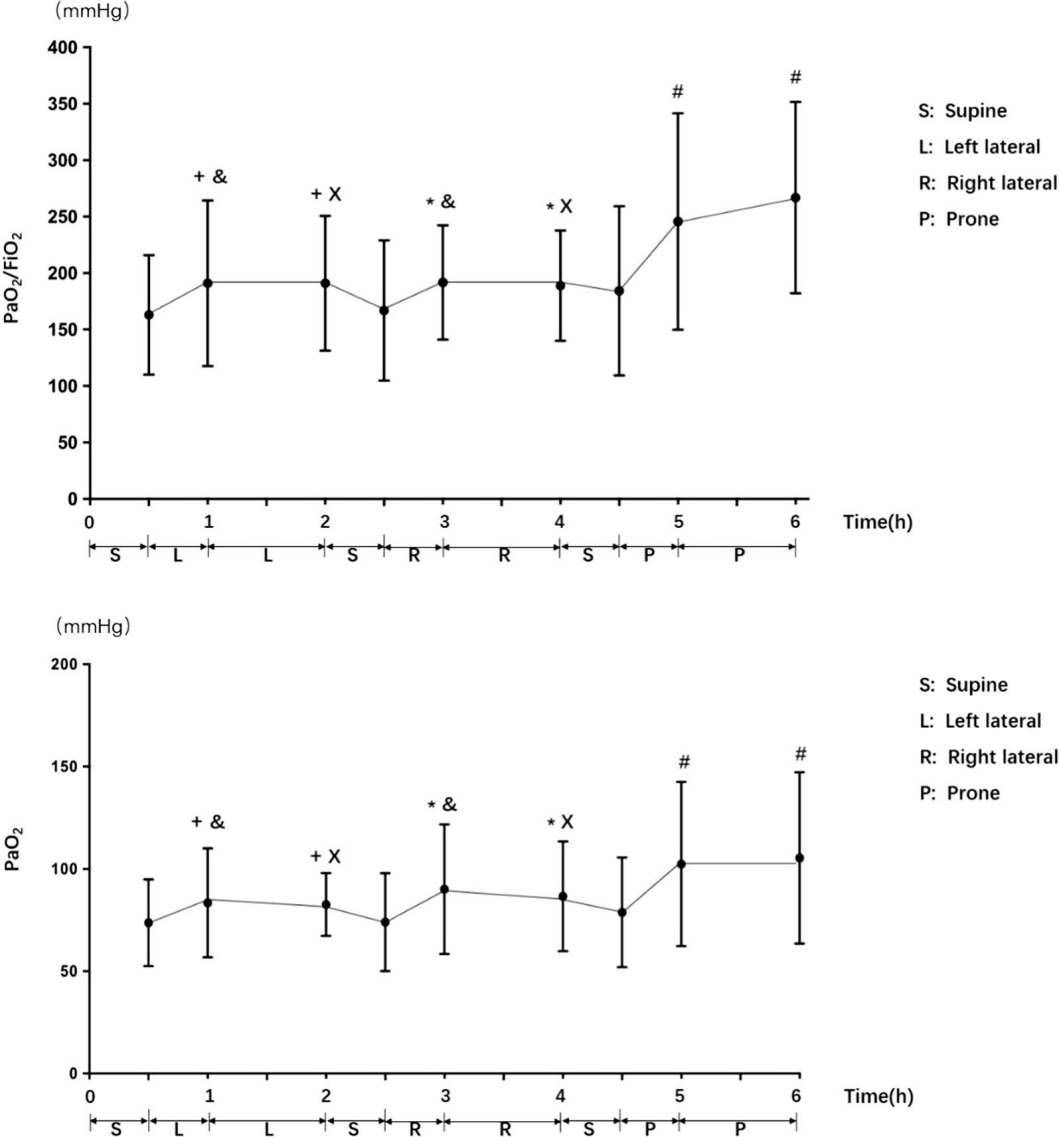
PaO_2_/FiO_2_ and PaO_2_ improved in left lateral, right lateral, and prone position compared with supine position (^+^*P* < 0.05 vs supine 0.5 h, ^*^P < 0.05 vs supine 2.5 h, ^#^*P* < 0.05 vs supine 4.5), and the improvement degree of PaO_2_/FiO_2_ and PaO_2_ in left and right lateral position was less than that of prone position (^&^*P* < 0.05 vs supine prone 5 h, ^x^*P* < 0.05 vs supine prone **6h)**

SAS v9.4 software was used for statistical analysis. Normally distributed quantitative data are described by the mean ± standard deviation (x ± s). The *P* values of pairwise comparisons were corrected by the Bonferroni method. The results showed that PaO_2_/FiO_2_ and PaO_2_ improved in the left lateral, right lateral and prone positions compared with the supine position. There was no significant difference between the left and right lateral positions, and it was independent of dominant side decubitus, but the improvement degree of PaO_2_/FiO_2_ and PaO_2_ was less than that of the prone position. There was no significant difference in oxygen saturation or respiration rate among different decubitus positions.

In conclusion, in HFNC patients with severe COVID-19, alternating left and right lateral positions improved oxygenation function. When the prone position is not tolerated for long periods of time, prone and lateral position alternating can be used to improve oxygenation.

## Data Availability

The datasets used and analyzed during the current study are available from the corresponding authors on reasonable request.
